# QuickPIV: Efficient 3D particle image velocimetry software applied to quantifying cellular migration during embryogenesis

**DOI:** 10.1186/s12859-021-04474-0

**Published:** 2021-12-04

**Authors:** Marc Pereyra, Armin Drusko, Franziska Krämer, Frederic Strobl, Ernst H. K. Stelzer, Franziska Matthäus

**Affiliations:** 1 Frankfurt Institute for Advanced Studies (FIAS) and Goethe Universität Frankfurt am Main, Ruth-Moufang-Straße 1, 60438 Frankfurt am Main, Germany; 2 Buchmann Institute for Molecular Life Sciences (BMLS), Max-von-Laue Straße 15, 60438 Frankfurt am Main, Germany; 3 Heidelberg University Hospital, Im Neuenheimer Feld 410, 69120 Heidelberg, Germany

**Keywords:** Particle image velocimetry, Light-sheet fluorescence microscopy, Collective cell migration, Julia, 3D image analysis, Tribolium castaneum

## Abstract

**Background:**

The technical development of imaging techniques in life sciences has enabled the three-dimensional recording of living samples at increasing temporal resolutions. Dynamic 3D data sets of developing organisms allow for time-resolved quantitative analyses of morphogenetic changes in three dimensions, but require efficient and automatable analysis pipelines to tackle the resulting Terabytes of image data. Particle image velocimetry (PIV) is a robust and segmentation-free technique that is suitable for quantifying collective cellular migration on data sets with different labeling schemes. This paper presents the implementation of an efficient 3D PIV package using the Julia programming language—quickPIV. Our software is focused on optimizing CPU performance and ensuring the robustness of the PIV analyses on biological data.

**Results:**

QuickPIV is three times faster than the Python implementation hosted in openPIV, both in 2D and 3D. Our software is also faster than the fastest 2D PIV package in openPIV, written in C++. The accuracy evaluation of our software on synthetic data agrees with the expected accuracies described in the literature. Additionally, by applying quickPIV to three data sets of the embryogenesis of *Tribolium castaneum*, we obtained vector fields that recapitulate the migration movements of gastrulation, both in nuclear and actin-labeled embryos. We show normalized squared error cross-correlation to be especially accurate in detecting translations in non-segmentable biological image data.

**Conclusions:**

The presented software addresses the need for a fast and open-source 3D PIV package in biological research. Currently, quickPIV offers efficient 2D and 3D PIV analyses featuring zero-normalized and normalized squared error cross-correlations, sub-pixel/voxel approximation, and multi-pass. Post-processing options include filtering and averaging of the resulting vector fields, extraction of velocity, divergence and collectiveness maps, simulation of pseudo-trajectories, and unit conversion. In addition, our software includes functions to visualize the 3D vector fields in Paraview.

## Background

Cellular migration in multi-cellular organisms often involves tissues or groups of cells that maintain stable or transient cell-cell contacts to preserve tissue integrity, sustain spatial patterning, or to enable the relocation of non-motile cells [1]. This phenomenon is generally known as collective cell migration, and it plays important roles in developmental processes, such as gastrulation or neural crest migration [2, 3], as well as in wound closure and cancer invasion [4]. Studies of collective cell migration on 2D cell cultures only partially reflect the physiology and architecture of in vivo tissues. Three-dimensional systems—such as model organisms, spheroids or organoids—are preferable, as they maintain physiological cell structures, neighborhood interactions, or mechanical extracellular properties, which have been recognized to play a role in regulating collective cellular migration [5–7]. Besides confocal fluorescence microscopy, light-sheet fluorescence microscopy (LSFM) has become one of the preferred techniques for three-dimensional imaging of biological samples, owing to its fast acquisition times, excellent signal-to-noise ratios, high spatial resolutions [8, 9], and low phototoxicity and photobleaching levels [10]. LSFM has been used to generate 3D time-lapse recordings of the complete embryonic morphogenesis of multiple model organisms [11, 12]. Based on light-sheet illumination, novel and improved imaging techniques are continuously being developed. For example, SCAPE (swept confocally-aligned planar excitation) microscopy offers more control over the viewing angle of the sample [13], while SVIM (selective volume illumination microscopy) dramatically increases acquisition times by dilating the light-sheet, at the expense of spatial resolution [14]. High temporal and spatial resolutions can be achieved with lattice light-sheet microscopy, where a combination of ultrathin light sheets and structured illumination are used [15]. The two last-mentioned techniques are particularly promising for resolving cellular migration and tissue rearrangements during quick morphogenetic events.

In order to quantify collective cellular migration in dynamic 3D biological data sets, we developed quickPIV, a free and open-source particle image velocimetry (PIV) package that offers fast and robust 3D, as well as 2D, PIV analyses. While several free and open-source 2D PIV software are readily available [16–19], the same is not true for 3D implementations. To the best of our knowledge, the Python implementation hosted in openPIV is the only other free and open-source PIV package that supports 3D analyses [18]. The fastest implementation in openPIV, however, corresponds to a 2D PIV implementation written in C++. Nevertheless, maintenance of this version was stopped in favor of the high-level and productive environment of its Python counterpart. In order to maximize performance without sacrificing productivity, our software is written in Julia, a modern programming language with high-level syntax similar to Python or Matlab that compiles to highly efficient code on par with C programs [20]. This choice is motivated by the high data volumes of 3D time-lapse recordings, which makes the analysis of multiple data sets computationally very expensive. For instance, a single sequence of 3D images of a developing embryo can easily reach data sizes of several Terabytes. Hence, the design principles of Julia enabled us to prioritize the CPU performance of quickPIV, and together with further optimizations, made it possible to reduce the processing speed of a pair of 3D volumes to several seconds.

The next subsection introduces PIV and discusses the strengths and limitations of applying PIV on biological samples. This is followed by a detailed description of the pipeline and the features implemented in quickPIV. The evaluation of our software includes a performance comparison to the C++ (2D) and Python (2D and 3D) implementations hosted in openPIV, as well as the accuracy evaluation of quickPIV on synthetic data. Furthermore, we analyze the ability of quickPIV to characterize migration patterns on three 3D time-lapse data sets of the embryonic development of the red flour beetle *Tribolium castaneum* [21, 22]. This is done by (1) simulating known translations on a 3D volume of *T. castaneum*, (2) validating the obtained vector fields against well-known migration patterns during the gastrulation of *T. castaneum*, and (3) by comparing the robustness of quickPIV on an embryo expressing both actin and nuclear molecular markers.

### Particle image velocimetry

Particle image velocimetry is a segmentation-free technique developed and established in the field of fluid dynamics to obtain displacement fields describing the motion of small tracer particles suspended in a flowing medium [23]. If the density of seeding particles is not exceedingly high [24], the motion of each suspended particle can generally be recovered through particle tracking velocimetry (PTV) [25]. PTV is analog to single-cell tracking, requiring the segmentation of all particles in two consecutive recordings before establishing one-to-one correspondences between the particle positions. While the size and seeding density of the tracer particles in hydro- and aerodynamic PIV experiments can be tuned [26], the segmentability of biological samples is challenged by factors with no or limited experimental control. For example, cell segmentation is hindered by low contrast of the molecular marker, irregular cell morphologies, or high cell densities. Instead of detecting and tracking individual objects, PIV relies on cross-correlation to find the translation that best aligns the intensity patterns contained inside any given sub-region between two consecutive recordings. Vector fields are generated by extracting displacement vectors from multiple sub-regions across the input data [23].

The accuracy of PIV on biological data is mostly explained by the strengths and limitations of cross-correlation. In short, cross-correlation is a pattern-matching operation that is suitable for finding translations of the intensity distributions contained in two successive recordings [27]. Therefore, PIV is appropriate for quantifying collective cell migration, which is dominated by a common translation of the migrating group of cells. Moreover, the pattern-matching nature of cross-correlation extends the application of PIV to non-segmentable data sets, including unstained samples or those stained with any persistent intra-cellular marker. PIV has been used to quantify cell migration in 2D model systems, such as wound healing assays [28], tumor invasion [29, 30], skin patterning [31] and others [32–34]. Conversely, cross-correlation is challenged by transformations other than translations, such as rotations, shears or deformations. High temporal resolutions alleviate the contribution of these transformations by approximating them to local translations. Uncoordinated cellular migration also reduces the similarity of intensity patterns between successive recordings, which degrades the accuracy of PIV. However, if the cells are sufficiently different from each other such that they are unambiguously detected by cross-correlation, a PIV analysis matching the size of the cells can be used to effectively track the movement of independently migrating cell [35, 36].

## Implementation

**Fig. 1:**
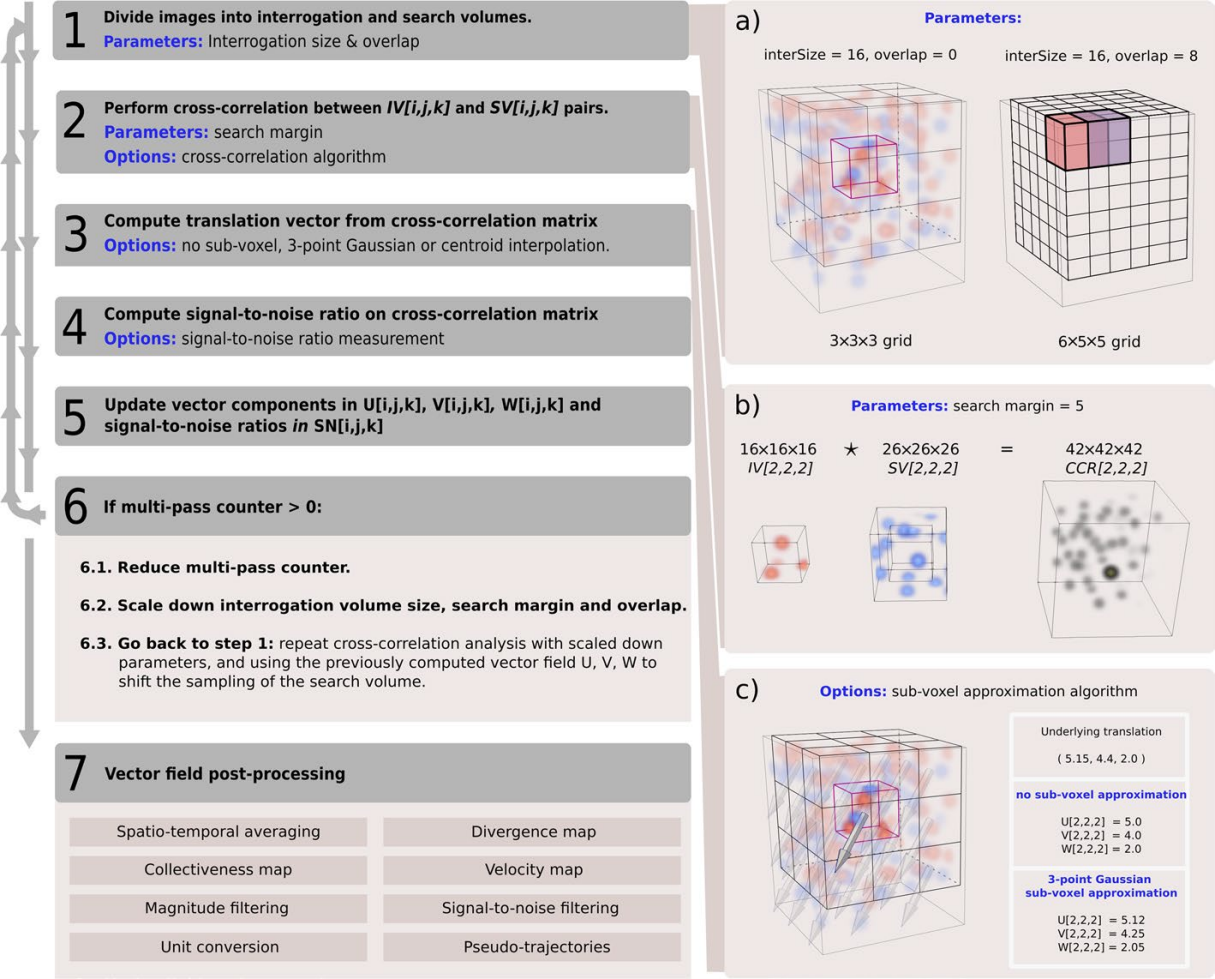
QuickPIV pipeline The PIV analysis starts by subdividing the input volumes, $V_{t}$ and $V_{t+1}$, into a grid of cubic interrogation, *IV*, and search volumes, *SV*. Cross-correlation is performed between each *IV*[*i*, *j*, *k*] and *SV*[*i*, *j*, *k*] pair, and a displacement vector, (*u*[*i*, *j*, *k*], *v*[*i*, *j*, *k*], *w*[*i*, *j*, *k*]), is computed from each cross-correlation matrix through the position of the maximum peak relative to the center of the cross-correlation matrix. The computed vector components are added to the *U*, *V* and *W* matrices. Optionally, signal-to-noise ratios are computed from each cross-correlation matrix and added to *SN*. If multi-pass is used, the cross-correlation analysis is repeated at progressively lower scales, which is achieved by scaling down the *interrogation size*, *overlap* and *search margin* parameters at each iteration. During multi-pass, previously computed displacements offset the sampling of the search volumes, effectively refining the computed displacements at each iteration. In order to post-process the PIV-computed vector fields, quickPIV currently implements: signal-to-noise and vector magnitude filtering, space-time averaging, divergence maps, velocity maps, collectiveness maps, pseudo-trajectories and unit conversion. (a) Left, two $60\times 50 \times 50$ voxel volumes are overlaid, with particles in $V_t$ shown in red, and particles in $V_{t+1}$ in blue. *Interrogation volume size* of $16 \times 16 \times 16$ voxels leads to $3\times 3 \times 3$ subdivision of non-overlapping interrogation and search volumes. Right, with 50% *overlap* the grid subdivision size is $6 \times 5 \times 5$. (b) Example of 3D cross-correlation between *IV*[2, 2, 2] and *SV*[2, 2, 2]. The use of a *search margin* of 5 voxels is illustrated, enlarging the search volume by 5 voxels in all directions. (c) Example of displacement computation. For clarity, this example portrays low particle densities and big particle radii, which results in sub-optimal accuracy of the 3-point Gaussian sub-voxel approximation

This section outlines the three-dimensional PIV pipeline implemented in quickPIV. The workflow of a PIV analysis in quickPIV is illustrated in Fig. 1. This figure shows input volumes containing Gaussian particles to ease the visualization of the underlying translation. To accommodate all possible labeling schemes of biological samples, we generally refer to structures or intensity patterns in the analyzed data.

The input to a 3D PIV analysis is a pair of 3D volumes taken at consecutive time points, $V_{t}[x,y,z]$ and $V_{t+1}[x,y,z]$, where (*x*, *y*, *z*) corresponds to the unique 3D coordinates of each voxel. Both input volumes are assumed to have the same dimensions. First, $V_{t}$ is subdivided into a 3D grid of cubic sub-regions known as interrogation volumes, *IV*[*i*, *j*, *k*], each specified by its position in the grid, (*i*, *j*, *k*). The dimensions of the grid subdivision are determined by the interrogation volume size and the overlap between adjacent interrogation volumes, see Fig. 1a. For each interrogation volume, a corresponding search volume, *SV*[*i*, *j*, *k*], can be defined in $V_{t+1}$.

Structures moving inside *IV*[*i*, *j*, *k*] by a translation ${\mathbf {s}} = (s_x, s_y, s_z)$ are expected to be found $\Vert {\mathbf {s}}\Vert$ voxels away in the direction of the translation in *SV*[*i*, *j*, *k*]. The underlying translation, ${\mathbf {s}}$, of the structures contained in *IV*[*i*, *j*, *k*] and *SV*[*i*, *j*, *k*] is recovered through a cross-correlation analysis [27]. The cross-correlation between a pair of interrogation and search volumes results in a 3D cross-correlation matrix. In the absence of other transformations, the vector from the center to the maximum peak of the cross-correlation matrix reflects the underlying translation of the structures contained in *IV*[*i*, *j*, *k*] and *SV*[*i*, *j*, *k*]. The structures visible in *IV*[*i*, *j*, *k*] may move outside the borders of the corresponding *SV*[*i*, *j*, *k*]. This is known as out-of-frame loss, and it limits the ability of cross-correlation to match the spatial intensity distributions between the pair of interrogation and search volumes. This can be compensated by enlarging the search volumes by a given margin along all dimensions, designated as search margin in quickPIV. The search margin should not be much larger than the expected translation strength of the structures, as enlarging the search volumes comes at the expense of performance. Figure 1b depicts the cross-correlation of the central interrogation and search volumes in Fig. 1a, including a search margin of 5 voxels around the search volume.

By computing a displacement vector for each pair of interrogation and search volumes, PIV analyses generate a vector field that describes the velocity distribution of the structures contained in the input volumes. The components of the PIV-computed vector field are returned separately in three 3D matrices: *U*, *V* and *W*. It should be noted that the resolution of the final vector field is decided by the size of the interrogation volumes and their overlap, which determine the grid subdivision of $V_{t}$ and $V_{t+1}$. Multi-pass is implemented to overcome this trade-off between resolution and the interrogation size of the PIV analysis.

### Cross-correlation

The cross-correlation of two one-dimensional real-valued functions is defined as:

1 $$\begin{aligned}{}[ f \star g ](s) = \int _{-\infty }^{\infty } f(x)g(x + s) \mathrm {d}x \ , \end{aligned}$$

where *s* has the effect of shifting *g*(*x*) along the x-axis. Cross-correlation involves computing the dot product of *f*(*x*) and $g(x+s)$ for all possible values of *s*. Since the dot product entails a basic measure of similarity, the value of *s* that achieves the highest dot product represents the translation that best aligns the two functions.

The form of cross-correlation in Eq. (1) is known as spatial cross-correlation. Discrete implementations of spatial cross-correlation have a 1D complexity of $O(N^2)$. Taking advantage of the convolution theorem, cross-correlation can be computed in the frequency domain through Fourier transforms of *f*(*x*) and *g*(*x*):

2 $$\begin{aligned} f \star g = {\mathcal {F}}^{-1}\{ \overline{{\mathcal {F}}\{f\}} \cdot {\mathcal {F}}\{g\}\} \ , \end{aligned}$$

where ${\mathcal {F}}$ and ${\mathcal {F}}^{-1}$ denote the Fourier and inverse Fourier transforms, respectively. Each Fourier and inverse Fourier transform in Eq. (2) can be computed efficiently with the Fast Fourier Transform (FFT) algorithm [37], which has a 1D complexity of $O( N \log {N} )$. Since Eq. (2) does not involve any operations with higher complexities than FFT’s, the overall complexity of 1D cross-correlation in the frequency domain is $O( N \log {N} )$. For this reason, cross-correlation in quickPIV is computed in the frequency domain. We rely on a Julia wrapper around the mature and optimized Fastest Fourier Transform of the West (FFTW) C library [38] to compute all Fourier and inverse Fourier transforms. FFTW implementations of FFT generalize to multi-dimensional data, enabling the efficient three-dimensional computation of cross-correlation.

To tackle the bias of the dot product towards high intensities, we implemented zero-normalized cross-correlation (ZNCC). Considering *IV* and *SV* as a pair of 3D interrogation and search volumes, ZNCC is calculated at each translation of *IV* by:

3 $$\begin{aligned} ZNCC[\mathbf{s }] = \sum _{x,y,z} \frac{ (IV[\mathbf{x }]-\mu _{IV})(SV[\mathbf{x } + \mathbf{s } ]-\mu _{SV}) }{ \sqrt{ \sum _{x,y,z}( IV[\mathbf{x }] - \mu _{IV} )^2 \sum _{x,y,z}( SV[\mathbf{x } + \mathbf{s }] - \mu _{SV} )^2 } } \, \end{aligned}$$

where **x** is a 3D index (*x*, *y*, *z*) running over all voxels of *IV*, **s** is the displacement vector $(s_x,s_y,s_z)$, and $\mu _{IV}$ and $\mu _{SV}$ are the average intensity values of the interrogation and search volumes, respectively. Zero-normalized cross-correlation is implemented efficiently in quickPIV following the work of Lewis, who noted that the numerator in Eq. (3) can be computed efficiently in the frequency domain, while each sum in the denominator can be calculated with eight operations from an integral array of the search volume [39].

To further improve the pattern-matching robustness of cross-correlation, quickPIV also offers normalized squared error cross-correlation (NSQECC). At each translation of *IV*, NSQECC is computed as [40]:

4 $$\begin{aligned} NSQECC[\mathbf{s }] = \sum _{x,y,z} \frac{ (IV[\mathbf{x }]-SV[\mathbf{x } + \mathbf{s } ])^2 }{ \sqrt{\sum _{x,y,z}(IV[\mathbf{x }])^2 \sum _{x,y,z}(SV[\mathbf{x } + \mathbf{s }])^2 } } \ , \end{aligned}$$

where **x** is a 3D index (*x*, *y*, *z*) running over all voxels of *IV*, and **s** is the displacement vector $(s_x,s_y,s_z)$. Following the example of [39], Eq. (4) is implemented efficiently in quickPIV by expressing the numerator and denominator in terms of three components: $\sum (IV[\mathbf{x }])^2$, which is constant, $\sum (SV[\mathbf{x }+\mathbf{s }])^2$, which is computed efficiently for each translation from an integral array, and $-2\sum (IV[\mathbf{x }]SV[\mathbf{x }+\mathbf{s }])$, which can be computed as an unnormalized cross-correlation in the frequency domain. For convenience, quickPIV implements the inverse of Eq. (4), $1 / ( 1 + NSQECC[\mathbf{s }])$, to obtain a maximum peak at the translation that minimizes the differences between the interrogation and search volumes.

### Peak sub-voxel approximation

In order to detect non-integer translations, two sub-voxel interpolation methods are included in quickPIV: the centroid-based and the 3-point Gaussian sub-voxel approximations [41]. In both methods, sub-voxel refinements are computed by considering the direct neighboring values around the maximum peak of the cross-correlation matrix. The centroid-based sub-voxel refinements, $\Delta$, are computed by

5 $$\begin{aligned} \Delta [\mathbf{d }] = \frac{C[\mathbf{x }+\mathbf{d }] - C[\mathbf{x }-\mathbf{d }]}{C[\mathbf{x }+\mathbf{d }] + C[\mathbf{x }] + C[\mathbf{x }-\mathbf{d }]} \ , \end{aligned}$$

where *C* refers to the cross-correlation matrix, $\mathbf{x }$ are the voxel coordinates of the maximum peak in the cross-correlation matrix, and $\mathbf{d }$ is the standard basis vector for each dimension, e.g. (1, 0, 0) for the first dimension. Following the same notation, the 3-point Gaussian sub-voxel refinement of the integer displacement is given by

6 $$\begin{aligned} \Delta [\mathbf{d }] = \frac{ \ln { (C[\mathbf{x }+\mathbf{d }]) } - \ln { (C[\mathbf{x }-\mathbf{d }]) } }{ 2\;\ln {(C[\mathbf{x }+\mathbf{d }])} - 4\;\ln {(C[\mathbf{x }])} + 2\;\ln {(C[\mathbf{x }-\mathbf{d }])} } \ . \end{aligned}$$

To acquire sub-voxel precision, the interpolated $\Delta$ is added to the integer displacement vector from the maximum peak to the center of the cross-correlation matrix. QuickPIV defaults to the 3-point Gaussian sub-voxel approximation, which performs particularly well when the input volumes contain Gaussian particles, as the convolution of Gaussians produces another Gaussian distribution [42].

### Multi-pass

We implemented a multi-pass procedure to increase the accuracy of the PIV analysis and to extend its dynamic range, i.e., the range of detectable displacements. While a search margin can be added to increase the dynamic range of a standard PIV analysis, it does not eliminate the dependence on small interrogation volumes to achieve high resolutions, which limits the specificity and enhances the noise of the intensity patterns contained in the interrogation volumes [43]. Alternatively, high resolutions with good dynamic ranges can be achieved by combining large interrogation volumes with high overlaps. However, this approach is computationally expensive and increases the final resolution by adding redundancy between consecutive cross-correlation computations [44].

The multi-pass algorithm starts the PIV analysis with up-scaled interrogation and search volumes, followed by iterative rounds of PIV analyses with gradually smaller interrogation size and search volumes. Additionally, the displacements calculated during previous rounds are used to offset the sampling of the search volumes at future rounds [45]. The multi-pass factor *f* defines the number of total rounds that will be conducted. Therefore, multi-pass is enabled by setting *f* larger than 1. At each multi-pass round, the interrogation size, search margin and overlap parameters are scaled with respect to their user-defined values. The value of these parameters in each round *r* is computed as follows:

7 $$\begin{aligned} \kappa _r = ( 1 + f - r )\ *\ \kappa _0 \ , \end{aligned}$$

where $\kappa _0$ designates the user-defined value for interrogation size, search margin or overlap, $\kappa _r$ is the up-scaled value of these parameters at round *r*, and *f* is the multi-pass factor. The final round is performed with a factor of 1, i.e., the initial interrogation sizes.

### Post-processing

Some of the post-processing features explained below include local information around the vector being processed. In such cases, a square (2D) or cubic (3D) region is sampled around each post-processed vector. For instance, $r_x$ and $r_y$ define a square area around an arbitrary vector in a 2D vector field, $v_{i,j}$, given by $L = \{ v_{i+r_x,j+r_y} \ | \ -r \le r_x \le r \ and \ -r \le r_y \le r \}$.

#### Filtering

A PIV-computed vector is considered unreliable if it was computed from a cross-correlation matrix containing multiple peaks with similar heights as the maximum peak. This reveals uncertainty about the underlying displacement, which might be caused by unspecific structures, background noise and/or loss of structure pairs [46, 47]. QuickPIV adopts the primary peak ratio, *PPR*, to measure the specificity of each computed vector,

8 $$\begin{aligned} \mathrm {PPR} = \frac{C_{\max1}}{C_{\max2}} \ , \end{aligned}$$

where $C_{\rm max}{1}$ is the height of the primary peak in the cross-correlation matrix and $C_{\rm max}{2}$ is the height of the secondary peak. Vectors with high *PPR* values are considered to have high signal-to-noise ratios [48]. Therefore, quickPIV offers filtering of unreliable vectors by discarding those vectors with a *PPR* value lower than a given threshold, $th_{\rm PPR}$ [48].

Additionally, quickPIV includes both global and local filtering in terms of vector magnitudes. Currently, quickPIV offers low pass and high pass filters of vector magnitudes, which can be concatenated to perform band-pass filtering. Global magnitude filtering can also be performed on those vectors whose magnitude is more than a certain number of standard deviations away from the mean magnitude of the vector field. Local magnitude filtering is implemented by discarding vectors whose magnitude is at least *n* standard deviations away from the mean magnitude, computed in a radius *r* around each vector.

All filtering functions in quickPIV accept an optional argument that is used to determine the replacement scheme of the filtered vectors. Currently, quickPIV offers three replacement functions: zero-replacement, mean replacement and median replacement. The former sets all components of the filtered vectors to zero. Both the mean and median replacement schemes are parametrized by the radius of the neighboring region used to compute the mean or median vector.

#### Spatial and temporal averaging

Spatial and spatio-temporal averaging of the computed vector fields are included in quickPIV. Spatial averaging depends on one parameter: the radius, $r_s$, of the considered neighboring region around each vector. Different radii for each dimension can be provided by passing an array of values, $[ r_x, r_y, r_z ]$. Spatio-temporal averaging considers two parameters: the averaging radius in space and the number, $n_t$, of adjacent vectors along the time axis considered in the temporal averaging, e.g. $\{ v_{i,j,k,t+r} | -n_t \le r \le n_t \}$.

#### Similarity-selective spatial averaging

Spatial averaging tends to dissolve vectors adjacent to the background and creates artifactual vectors in regions containing dissimilar vectors. A similarity-selective spatial averaging has been developed to overcome these limitations, and to enhance the visualization of collective migration. Two vectors are considered to be similar if they point in the same direction, which is established if their normalized dot product is greater than a user-defined threshold. Given any vector in the PIV-computed vector field, $\mathbf {v}[i,j,k]$, an average vector is built by considering only those neighboring vectors at a radius *r* that are similar to $\mathbf {v}[i,j,k]$. The averaged vector is then normalized to unit length, and its magnitude is further re-scaled by the ratio between the number of similar neighboring vectors and the total number of neighboring vectors. Therefore, the effect of similarity-selective averaging is to average the direction of each vector among similar neighboring vectors, and to re-scale the magnitude of each vector by the local collectiveness.

#### Mappings

QuickPIV provides functions for extracting several relevant quantities from the PIV-computed vector fields. Velocity maps are generated by returning the magnitude of each vector from a given vector field. QuickPIV implements convergence/divergence mappings to detect the presence of sinks and sources in the PIV-computed vector fields. This is done by generating a cube of normalized vectors that either converge (sink) or diverge (source) from the center of the cube, and cross-correlating this cube with the normalized vector field. This mapping is parametrized by the size of the cube, which determines the scale of the convergence/divergence map. Collectiveness maps are built by computing the number of neighboring vectors at a radius *r* from each vector in the vector field $v_{i,j}$ whose normalized dot product is greater than a threshold.

#### Pseudo-trajectories

Pseudo-trajectories can be generated with quickPIV to visualize the approximate paths of cells and tissues from the PIV-computed vector fields. When computing pseudo-trajectories, a user-defined number of particles is randomly distributed within the dimensions of the vector field. The position of each particle is rounded to integer coordinates in order to sample a displacement from the vector field, which shifts the particle from its current position. By repeating this process, a three-dimensional path is obtained for each simulated particle. It is possible to constrain the computation of pseudo-trajectories to a period of interest by specifying the start and end time points. Moreover, spatially interesting regions can be selected by specifying the spatial range over which to initialize the positions of the particles.

#### Conversion to physical units

Last but not least, to convert voxel displacements into physically meaningful velocities both the frame rate and the physical units of each voxel dimension need to be taken into account. These values can be provided during the creation of the PIV-parameter object and quickPIV will automatically re-scale the resulting vector field after the analysis.

### QuickPIV accuracy evaluation

The correct implementation of a PIV analysis depends on its ability to detect translations. Accordingly, the accuracy of quickPIV is assessed by generating pairs of artificial images and volumes containing synthetic particles related by a known translation. Synthetic particles are rendered according to [49]. The bias and random errors are computed to evaluate the agreement of quickPIV predictions to the known translations [49]:

9 $$\begin{aligned}&\epsilon _{\rm bias} = \frac{1}{n}\sum _{i=1}^{n} | d_{PIV,i} - d_{\rm true} | \end{aligned}$$

10 $$\begin{aligned} \epsilon_{\rm rand} = \sqrt{ \frac{1}{n} \sum _{i=1}^{n} {(d_{{\rm PIV},i}-\overline{d_{\rm PIV}})^2}} \end{aligned}$$

where $d_{{\rm PIV},i}$ is the $i^{\mathrm {th}}$ PIV-computed displacement, $d_{\rm true}$ is the known translation, $\overline{d_{\rm PIV}}$ is the average PIV-computed displacement and *n* is the number of repeats. The bias and random errors represent the accuracy and the precision of quickPIV’s approximation of the underlying translation, respectively. The effect of the following parameters on the accuracy of quickPIV are evaluated, both in 2D and 3D: interrogation size, particle density, particle diameter, 3-point Gaussian sub-pixel approximation and the use of a search margin to correct for out-of-frame loss.

### QuickPIV performance evaluation

The performance of our software is evaluated by comparing the execution times of quickPIV with those of the C++ and Python implementations hosted in openPIV. First, we analyzed the time required to compute cross-correlation in the frequency domain with the three packages. By comparing the execution times of quickPIV and the C++ implementation, we can determine whether calling the FFTW C-library from Julia adds any noticeable overhead compared to C++. Since the Python implementation uses the NumPy library to compute the Fourier and inverse Fourier transforms, this test also reveals any performance differences between FFTW and NumPy. On the other hand, we compare the execution time of complete 2D and 3D PIV analyses between the three PIV packages. The set of parameters used in these PIV analyses are listed in the description of Table 1.

**Fig. 2:**
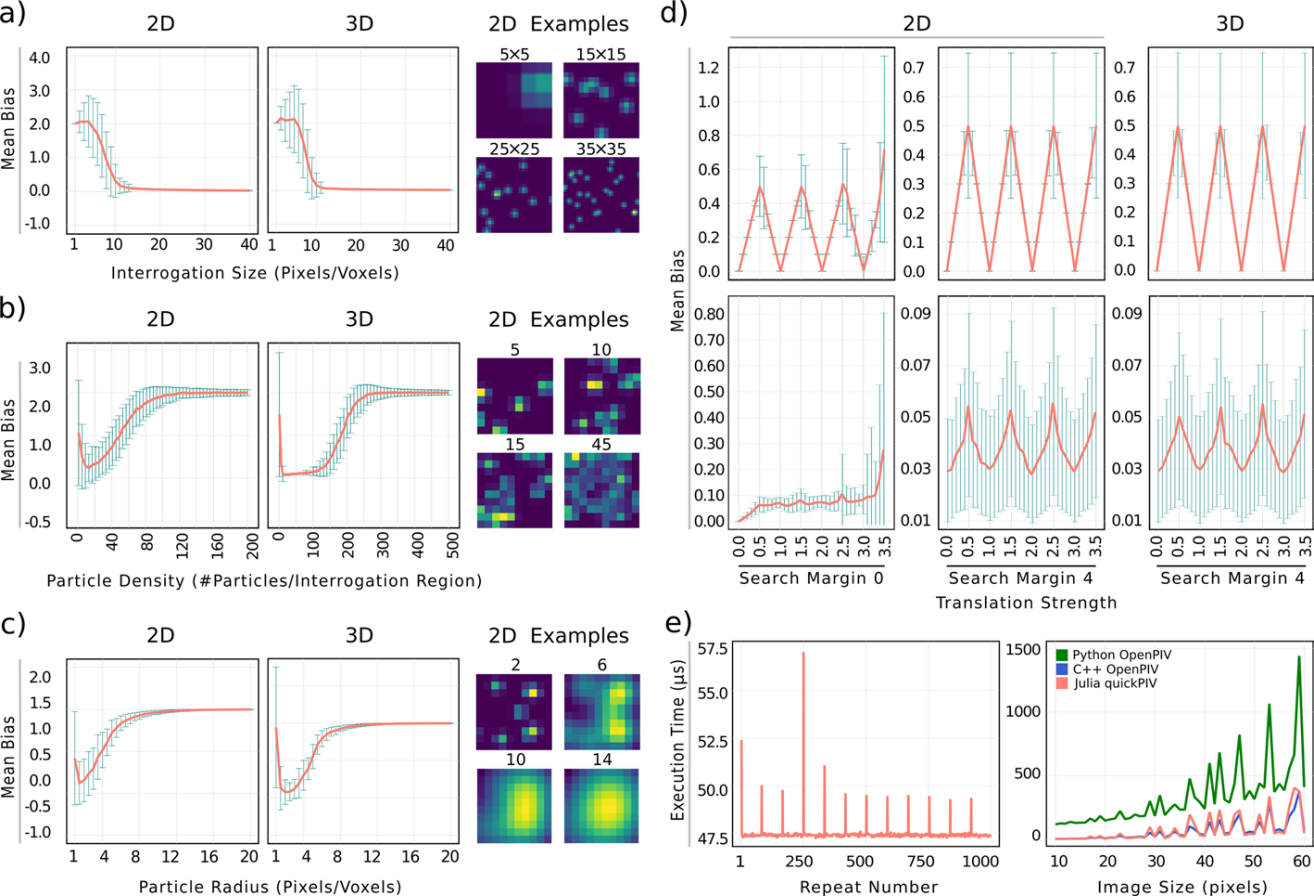
Accuracy and performance evaluations of quickPIV. **a**–**d** Mean biases (red lines) and random errors (green error bars) of unnormalized PIV applied to synthetic data containing particles shifted by homogeneous translations. **a** PIV errors are reduced by increasing interrogation size. As illustrated under the 2D examples, the intensity patterns contained in small interrogation areas (5$\times$5 pixels) display unspecific structures, and are more susceptible to out-of-frame loss. The 2D analyses were performed on 200$\times$200 pixel images containing 5k particles, and 3D analyses on 200$\times$200$\times$200 voxel volumes with 100k particles. **b** Particle densities of around 15 particles per interrogation region minimize PIV errors. Low particle count are susceptible to out-of-frame loss, while high particle densities degrade PIV accuracies by producing uniform intensity patterns. Interrogation size during this evaluation was 10$\times$10 pixels and 10$\times$10$\times$10 voxels. **c** Particle sizes of 1-2 pixels achieve optimal PIV accuracies. The 2D examples show that large particle radii blur the intensity pattern inside the interrogation regions, reducing the pattern complexity. **d** Top, PIV accuracy under non-integer translations oscillates between 0.0 and 0.5. Bottom, with 3-point Gaussian interpolation, errors are reduced by an order of magnitude. The leftmost figures show a slight loss of accuracy due to out-of-frame loss as the translation strength increases. Adding a search margin greater than the translation strength completely compensates for this effect. **e** Left, execution times distribution of 1000 FFT computations on input images of $40 \times 40$ pixels. Background processes sporadically slow down FFT execution. Right, comparison of 2D FFT performance between Julia, C++ and Python for increasing input sizes. Julia and C++ calls of FFTW are equally fast, while the FFT implementation in NumPy is approximately three times slower. The execution time of FFT spikes when the input sizes are prime numbers, e.g. 23, 29 or 43

For the sake of using a common benchmarking pipeline, language-specific packages for measuring the execution times are avoided. Each execution time measurement shown in Fig. 2e corresponds to the minimum execution time from 1000 repeated measurements. Taking the minimum execution time filters out random delays originating from background processes [50]. The left panel in Fig. 2e illustrates the interference of background processes in the distribution of 1000 execution measurements of FFT cross-correlation. All measurements presented below were performed on a machine with an Intel Core i5-8300H processor $4\times 2.3$ GHz. All PIV analyses were executed on a single thread.

### QuickPIV on the embryogenesis of *Tribolium castaneum*

To test the accuracy of quickPIV on biological data, we analyzed three 3D time-lapse data sets of the embryonic development of *T. castaneum*: (1) two embryos from a hemizygous transgenic line that ubiquitously expresses nuclear-localized mEmerald and (2) one embryo from a double hemizygous transgenic line that expresses nuclear-localized mRuby2 ubiquitously and actin-binding Lifeact-mEmerald only in the serosa [22]. Using LSFM, the embryos were recorded at intervals of (1) 30 minutes or (2) 20 minutes along 4 directions in rotation steps of 90$^{\circ }$ around the anterior-posterior axis in (1) one or (2) two fluorescence channels [21]. The four directions were fused according to Preibisch et al. [51] to generate evenly illuminated volumes with isotropic resolution. The fused volumes were cropped to $1000\times 600 \times 600$ voxels (height,width,depth), the embryos were manually placed in the center of the volumes and their anterior–posterior axis was manually aligned with the vertical axis.

**Fig. 3:**
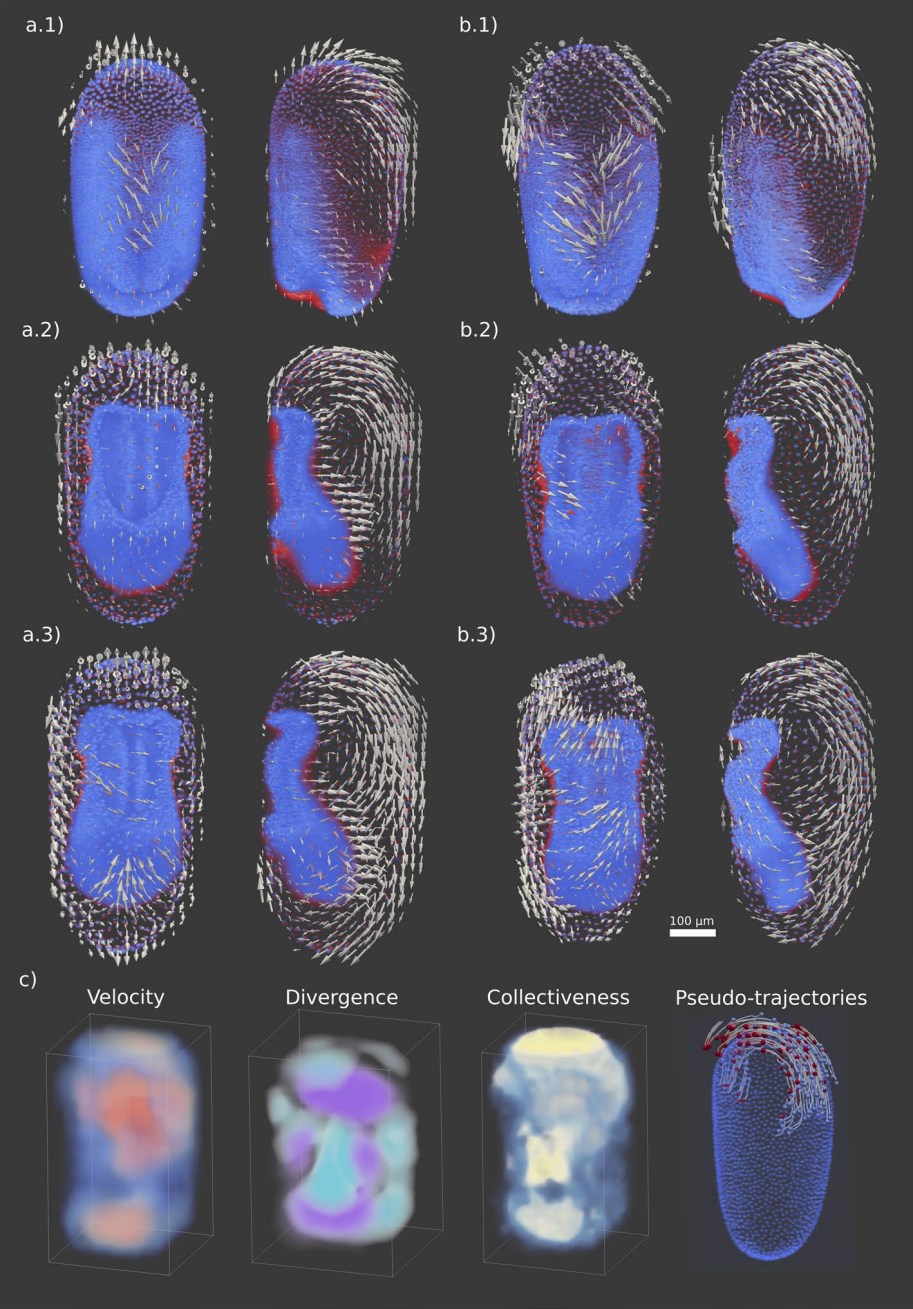
3D PIV analysis on the embryogenesis of two *T. castaneum* embryos. Each vector field in **a**. 1–3 and **b**. 1-3 is plotted on top of the two volumes it was computed from, where the red signal corresponds to the initial time point and blue intensities belong to the consecutive time point. A few spurious vectors obtained on the background due to the fluorescence bleeding from the embryo were manually curated. Embryos are shown from their ventral and lateral sides. **a-b.1** At the onset of gastrulation, serosa nuclei at the anterior end of both analyzed embryos collectively spread towards the dorsal side of the embryos. Moreover, the central and posterior regions on the ventral side undergo coordinated condensation movements that will later give rise to the internalizing germband. **a-b.2** The wide-spread serosa cells over the anterior pole and the dorsal side engage in a highly coordinated movement of the tissue towards the posterior pole. Time points **a-b.3** are characterized by a highly collective flow of serosa cells towards the ventral side, leading to the emergence and closing of the serosa window. Serosa cells at the anterior pole, dorsal side and the posterior pole collectively migrate clock-wise towards the ventral midline, giving rise to a cell migration pattern resembling a vortex. **c** Exemplary post-processing analyses applied to the vector field shown in a.1. From left to right: velocity map showing higher velocities in red, divergence(purple)/convergence(cyan) map, collectiveness map displaying higher local collectiveness in yellow, and pseudo-trajectories at the anterior pole of the embryo in a.1) over 10 time points (5 h)

**Fig. 4:**
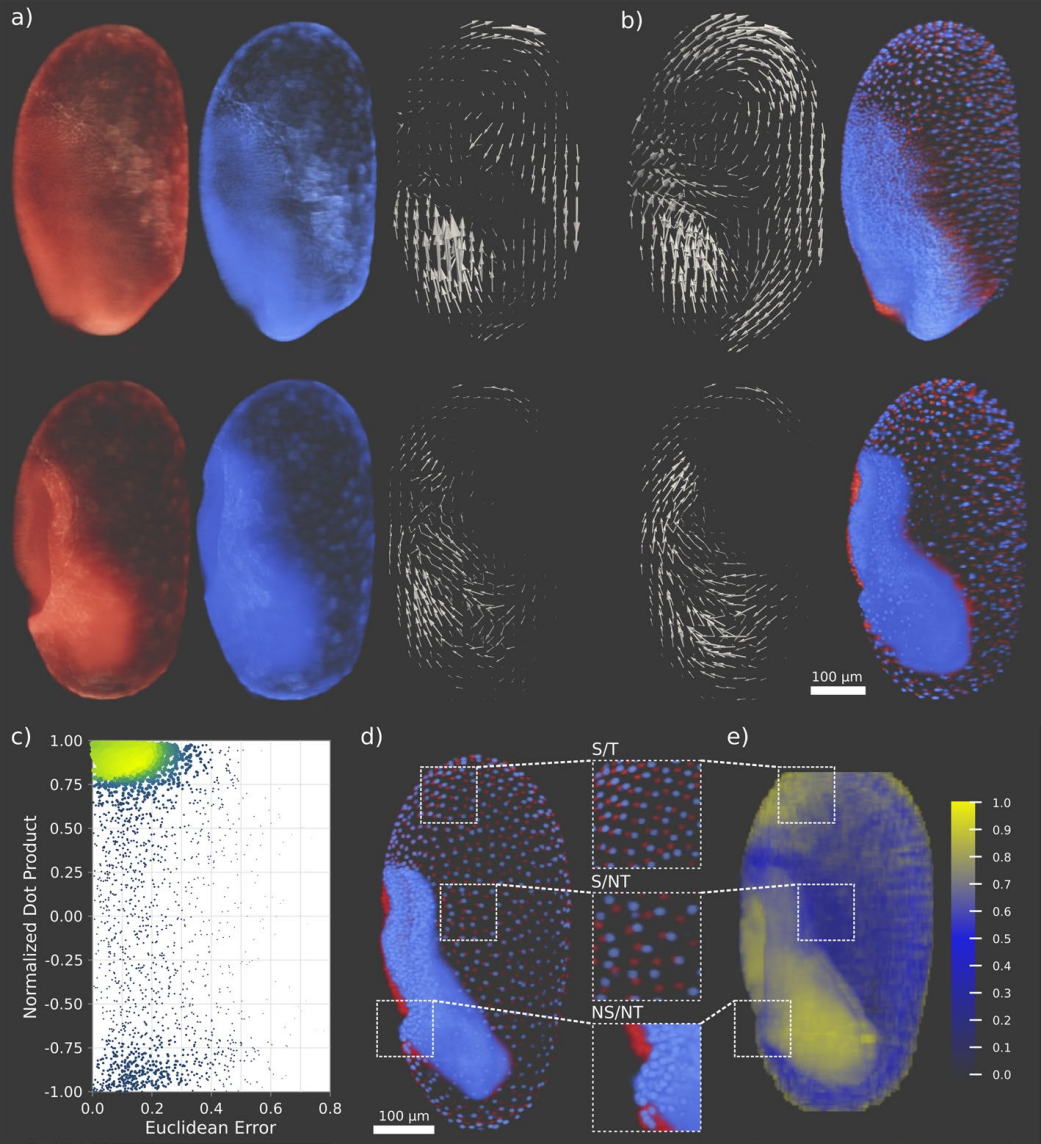
Validation of quickPIV on non-segmentable data sets. **a** PIV analyses were performed on the actin signal of a double hemizygous transgenic embryo before (top) and during (bottom) gastrulation. For each time point, the two consecutive volumes analyzed with PIV are shown in red and blue, next to the computed vector fields after similarity-selective spatial averaging. **b** PIV was also performed for the same time points on the nuclear signal, and the resulting similarity-selective averaged vector fields are shown next to the actin vector fields. **c** The orientation similarity between each pair of vectors in the two channels is computed through their normalized dot product. The Euclidean error between each pair of vectors is computed as well to measure the combined magnitude and direction differences between the vectors. The scatter plot of these two quantities shows that most vectors are clustered around a region of high normalized dot product and low euclidean error, indicating good agreement between the vector fields in (**a**) and (**b**). **d** Three patterns of cell migration can be distinguished in the *T. castaneum* data set (i): Segmentable and trackable (S/T), segmentable and non-trackable (S/NT) and non-segmentable and non-trackable (NS/NT) nuclei. The serosa consists of segmentable nuclei. While some regions are easily trackable, in others it is difficult to establish unambiguous correspondences of the nuclei between the two time points. High cell densities render nuclei in the gastrulating embryo non-segmentable, and therefore non-trackable. **e** Three-dimensional mapping of the height of the maximum peak of NSQECC at each interrogation area during the PIV analysis of the two volumes in (**d**). High values are achieved both in the segmentable and trackable and non-segmentable regions of the embryo, indicating that the interrogation and search patterns in these regions are well approximated by a translation and high PIV accuracies are expected

Three time points during gastrulation were analyzed with quickPIV in the two embryos of data set (i). Two time points of the double hemizygous transgenic line (ii) were analyzed in both channels, allowing to compare the vector fields obtained from the Lifeact-mEmerald actin signal with those from the nuclear-localized mRuby2 marker. The PIV analyses were performed on both data sets with NSQECC. The vector fields resulting from these analyses are shown in Figs. 3 and 4, post-processed with similarity-selective averaging with an averaging radius of 2 neighboring vectors and a similarity threshold of 0.5. The visualization of the embryo volumes and the computed vector fields has been done in Paraview 5.7.0.

## Results

The accuracy evaluation of quickPIV quantitatively reproduces the expected accuracies described in the PIV literature, attesting the correctness of our PIV implementation [52–55]. Our analysis shows a monotonic decrease of the total error (bias and random errors) with increasing interrogation sizes [55], reaching errors as low as $0.02 \pm 0.01$ pixels/voxels (Fig. 2a). This is the expected behavior in our synthetic tests, since all simulated particles are subjected to the same translation. Our results also agree on the presence of optimal values for both particle density and particle size [52]. It can be appreciated from the 2D examples included in Fig. 2b and c that high particle densities and large particle sizes generate diffuse images that can not be unambiguously matched by cross-correlation. Without sub-pixel/voxel interpolation, the PIV analysis cannot capture the decimal components of the simulated translations, shown in the top row of Fig. 2d [52, 53]. As described in the literature, the 3-point Gaussian sub-pixel approximation reduces this error by one order of magnitude (bottom row in Fig. 2d) [56]. Moreover, search margins are needed to counteract the out-of-frame errors induced by increasing translation (Fig. 2d, left panel). A search margin of 4 pixels/voxels (Fig. 2d, middle and right panels) completely compensates this effect for all simulated translations.

We performed an analogous accuracy analysis on the *T. castaneum* data set, where we quantified the accuracy of quickPIV in detecting know translations on one 3D volume in data set (i). We observed that diffuse and unspecific patterns in the embryo induce biases when using ZNCC. These biases are completely avoided by using NSQECC, which detects the underlying translation with 100% accuracy given a sufficiently large search margin (see Figure S1). We further analyzed the height distribution of the maximum cross-correlation peaks during the PIV analysis with NSQECC of two consecutive volumes of *T. castaneum*, shown in Fig. 4e. High peaks are found in the collectively migrating serosa cells at the anterior pole of the embryo, which we classify as segmentable and trackable (S/T), and in the non-segmentable and non-trackable (NS/NT) gastrulating embryo, Fig. 4d. These high peaks indicate that cellular migration in these regions is well approximated by a collective translation, and that the intensity patterns between the interrogation and search volumes are not deformed, rotated or sheared significantly. Non-collective migration of the serosa cells reduces the height of the NSQECC peaks in the central regions of the extraembryonic membranes, which we consider to be segmentable but not easily trackable (S/NT), since cell correspondences between the two time points can not unambiguously be assessed visually, Fig. 4d. A three-dimensional visualization of the maximum peak distribution in Fig. 4e is provided in Video S1.

Performance-wise, calling the FFTW C-library is equally efficient from Julia and C++ (see Fig. 2e, right). In contrast, the performance of the NumPy implementation of the FFT algorithm is three times slower than the one provided in the FFTW library. This performance difference is translated to the complete 2D and 3D PIV analyses of the PIV packages, where the Python implementation in openPIV is consistently three times slower than both the C++ implementation (2D) and quickPIV (2D and 3D), see Table 1. Our results also show that 2D PIV analyses are performed faster with quickPIV than with the C++ implementation in openPIV. Since both packages share the same cross-correlation performance, this difference can only be explained by compiler optimizations brought by Julia’s compilation pipeline, or by the ease of implementing good programming practices in Julia’s high-level environment. For instance, quickPIV avoids bound checks when possible, minimizes memory allocations by using in-place operations, and leverages SIMD (single instruction, multiple data) operations exposed by the Julia programming language.

**Table 1:** Performance evaluation of complete PIV analyses

	C++	Python	quickPIV
2D	63 ms	160.81 ms	50.42 ms
3D	-	59.72 s	18.09 s

2D analyses were performed on a pair of images of size 512$\times$512 pixel, with the following PIV parameters: FFT cross-correlation, interrogation size of 32 pixels, no search margin, overlap of 16 pixels, no multi-pass, 3-point Gaussian subpixel approximation and peak-to-peak signal-to-noise algorithm. 3D analyses were performed on volumes with dimensions 512$\times$512$\times$123 pixels, and the following PIV parameters: FFT cross-correlation, interrogation size of (49, 49, 11), no search margin, overlap of (14, 14, 3), no multi-pass, 3-point Gaussian sub-voxel approximation and peak-to-peak signal-to-noise algorithm. The reported measurement are the minimum execution time from 1000 and 100 repeats, for the 2D and 3D analysis respectively

From a practical standpoint, we found that performance of PIV analyses can be dramatically increased by subsampling the input volumes and removing the background interrogation areas from the PIV analysis. For example, a PIV analysis of two volumes of *T. castaneum* with the following parameters (interSize of 60 voxels, searchMargin of 0 voxels, overlap of 30 voxels and multi-pass factor of 2), while skipping interrogation volumes with a maximum intensity lower than 100, takes 29 minutes. After applying subsampling by a factor of 3, the analogous analysis on the subsampled data (interSize of 20 voxels, searchMargin of 0 voxels, overlap of 10 voxels and multi-pass factor of 2) takes 55 s to complete. The results shown in Figs. 3 and 4, which were obtained after subsampling the input volumes by a factor of three in all dimensions, are in full agreement with the same analyses performed without subsampling (Fig. S2). The spatial resolution in this data set was very high, which is necessary to discern smaller structures. For motion analysis a lower image resolution is sufficient to obtain the same results. Before subsampling images, we, however, advise to test the agreement between the PIV vector fields in the original and a subsampled image.

The application of quickPIV to the two *T. castaneum* embryos of data set (i) is shown in Fig. 3. The red and blue intensities correspond to the nuclear signal of the first and second input volumes, respectively, which aids in visualizing the underlying displacement of the nuclei between each pair of analyzed time points. The vector fields at the anterior regions of the embryos in Figs. 3a.1 and b.1 capture the underlying radially diverging pattern of cell migration towards the dorsal side of the embryo. Our PIV analyses also capture the coordinated condensation movement of the cells in the central and posterior regions, which will later give rise to the germband. These regions exhibit high cellular densities, challenging visual examination and rendering nuclei segmentation and tracking approaches unfeasible. Figure 3a.2 and b.2 are characterized by vastly coordinated movements of the wide-spread serosa cells over the anterior pole and along the dorsal side towards the posterior pole. Figures 3a.3 and b.3 depict a highly coordinated flow of serosa cells from the dorsal side over both, the posterior pole and the lateral equator, towards the ventral side, where they eventually give rise to the serosa window [57]. These observations are not only consistent with previous studies of collective cell migration during the gastrulation of *T. castaneum*, which were obtained through 1D PIV analyses [58] and manual 2D tracking of the extra-embryonic serosa cells [59, 60], but for the first time describe this process in 3D. Figure 3c illustrates the velocity, divergence/convergence and collectiveness mappings as well as some computed pseudo-trajectories on the anterior region of the embryo.

Finally, the results from the analysis of the double hemizygous transgenic line, (ii), demonstrate the robustness of quickPIV on non-segmentable data. The agreement of the vector fields on the anterior pole of the embryo (which is non-segmentable in the actin signal, segmentable in the nuclear signal and exhibits high degrees of collective cell migration in both channels) indicates that PIV accuracies are independent of the segmentability of the input data sets, Figs. 4a and b. A quantitative comparison of the PIV vector fields between the nuclear and the actin stained volumes shows a high degree of similarity. This is illustrated in the scatter plot shown in Fig. 4c, exhibiting a high density in the area of large dot products and small Euclidean errors. The similarity of the actin and nuclear vector fields in highly dense non-segmentable regions further underlines the robustness of quickPIV regardless of the labeling scheme of the data sets.

## Conclusions

QuickPIV represents a free and open-source solution for performing efficient and robust quantification of collective cellular migration in the increasingly popular 3D dynamic data sets in life sciences. Our software includes several well established PIV features, such as multi-pass and sub-voxel peak approximation, as well as post-processing functions and visualization of the 3D vector fields in Paraview.

To our knowledge, quickPIV is the only free PIV software that offers normalized squared error cross-correlation (NSQECC), which we found to be necessary for accurately describing collective cell and tissue migration in non-segmentable data sets. By using NSQECC, we could quantify collective cell migration from the non-segmentable and highly dynamic actin signal in a double hemizygous transgenic embryo of *T. castaneum*. The resulting vector fields were in complete agreement with previously published descriptions of the gastrulation movements in *T. castaneum*, and showed a strong correlation with the vector fields obtained from the nuclear signal of the same embryo. Moreover, the height distribution of the maximum cross-correlation peaks further indicates that NSQECC is robust to non-segmentable data.

The performance evaluation of quickPIV shows that our software is three times faster in 2D and 3D analyses than the Python PIV implementation in openPIV, and also faster than the 2D implementation written in C++. This performance advantage is only possible because of the design of the Julia programming language and the optimization possibilities that it provides. By considering subsampling and excluding unnecessary regions of the input data (such as empty background), the quickPIV analysis of a pair of 3D volumes can be reduced to several seconds. These speeds are compatible with real-time PIV analyses, enabling the integration of PIV pipelines into smart microscopy techniques. For example, vector fields obtained with quickPIV could be used to automatically detect the onset of developmental events and adjust the acquisition parameters accordingly, e.g. laser power or acquisition interval.

Overall, we believe that 3D PIV analyses will play an important role in understanding 3D biological processes as novel 3D imaging techniques are developed and adopted. For example, SVIM can already achieve up to 100$\times$ higher recording speeds than standard LSFM. Such high temporal resolutions increase the accuracy of PIV and make PIV the ideal solution for reliable and automated pipelines for quantifying collective cellular migration. However, the computational demands required to analyze such temporally resolved data sets can only be met by further optimizations of quickPIV’s performance. Therefore, future efforts will be directed towards adding multi-threading support and implementing our PIV analyses on a graphics card [61].

## Availability and requirements

Project name: quickPIV

Project home page: https://github.com/Marc-3d/quickPIV

Operating system(s): Platform independent

Programming language: Julia

Other requirements: Julia1.3.1 or higher

License: MIT License

Any restrictions to use by non-academics: None.

## Supplementary Information

**Additional file 1.** This file contains a video animating the rotation and slicing of the three-dimensional distribution of the maximum peak heights at each interrogation area during the PIV analysis using NSQECC of two 3D volumes of data set (i).

**Additional file 2.** This file contains **Figs. 1** and **2**. **Figure S1** reports the results form our synthetic accuracy evaluation on biological data, including the comparison between the accuracies obtained with ZNCC and NSQECC PIV analyses. **Figure S2** shows the same PIV analyses as Fig. 3, with the difference that no subsampling of the input volume was used to obtain the results in Figure S2.

## Data Availability

The data sets used and/or analyzed during the current study can be accessed through the following Zenodo 10.5281/zenodo.5504076.

## Declaration

### Software and code

The source code and instructions to run the evaluation scripts of quickPIV are available at Github: https://github.com/Marc-3d/quickPIV. The source code of the presented release of quickPIV, v1.0, can be accessed through: https://zenodo.org/record/5530573#.YVGyMX1CTBU.
