# Supplementary material for: QuickPIV: Efficient 3D particle image velocimetry software applied to quantifying cellular migration during embryogenesis
Source: BMC Bioinformatics. 2021 Dec 4;22:579. doi: 10.1186/s12859-021-04474-0 (PMC8642913; doi:10.1186/s12859-021-04474-0)
Supplement: Supplementary file 2 — Additional file 2. This file contains Figs. 1 and 2. Figure S1 reports the results form our synthetic accuracy evaluation on biological data, including the comparison between the accuracies obtained with ZNCC and NSQECC PIV analyses. Figure S2 shows the same PIV analyses as Fig. 3, with the difference that no subsampling of the input volume was used to obtain the results in Figure S2. [file 12859_2021_4474_MOESM2_ESM.pdf]

Supplementary Note on

QuickPIV: Efficient 3D particle image velocimetry software applied to quantifying cellular migration during embryogenesis

Marc Pereyra, Armin Drusko, Franziska Krämer, Frederic Strobl, Ernst H.K. Stelzer and Franziska Matthäus

1-. Evaluation of quickPIV on biological data

The accuracy evaluation shown in Figure 2 is indicative of the accuracy of PIV3D on datasets containing uniformly distributed Gaussian particles, like those generated for PIV analyses in fluid dynamic experiments. However, our main focus is the analysis of time-lapse recordings of dynamic biological processes, such as embryogensis. Therefore, we needed to assess the suitability of PIV on biological datasets, which contain heterogenous intensity patterns that are affected by the expression dynamics of the labelled molecules, the intracellular localization of the marker, cellular densities, cellular shapes, etc.

Quantifying errors in the *Tribolium Castaneum* dataset.

When analyzing biological data sets, there is no ground truth to compare our PIV result to. In order to generate a ground truth, we applied known translations to a volume of the *T. castaneum* dataset during gastrulation, and compared the vectors obtained with quickPIV to the known translations. In particular, we evaluated our software for translation strengths ranging between 0 and 10 voxels. The same translation strength is applied to each dimension, e.g. a translation strength of 4 voxels results in the 3D translation (4,4,4). Figure S1.b illustrates the test volume (red) and its shifted copy (blue) by a translation of 10 voxels in each direction. For each translation we record the ratio of correctly detected vectors, namely those whose Euclidean distance to the known translation, $\vec{T}$, is zero. The Euclidean distance between any given vector, $\vec{v}_i$, and $\vec{T}$ is given by:

$$Euclidean\, error = \sqrt{(\vec{v}_i - \vec{T}) \cdot (\vec{v}_i - \vec{T})}$$

where $\cdot$ signifies the dot product. We compare the accuracies of ZNCC and NSQECC PIV, with the following parameters: interrogation size of 20 voxels, overlap of 10 voxels, and no sub-voxel approximation. In addition, we repeat each PIV analysis for increasing values of search margin. Adding the search margins is expected to overcome out-ot-frame loss at high translations, hence increasing the accuracy of the analyses.

ZNCC vs NSQECC accuracy results

The plots in Figure S1a depict the accuracies of the ZNCC and NSQECC PIV analyses. Without search margin, SM0, ZNCC achieves 96% accuracy at a translation strength of zero, and the accuracy gradually decreases due to out-of-frame loss as the translation strength increases. Adding a search margin has the effect of lowering the maximum accuracy of ZNCC PIV to 67%, 62% and 54% for SM5, SM10 and SM20, respectively. In spite of this drop in the maximum accuracy, higher search margins mitigate further loss of accuracy due to out-of-frame loss as the translation strength increases. (Bottom) Without search margin, NSQECC PIV accuracies decrease from 100% to 8% as stronger translations are simulated. Adding search margins higher than the simulated translations (SM10 and SM20) completely remove out-of-frame loss, resulting in 100% accuracies for all translations.

Distribution of ZNCC errors

The fact that the accuracy with ZNCC PIV at SM0 is lower than 100%, together with the reduction of maximum accuracies with the introduction of search margins, points to the presence of biases of ZNCC on biological data. When using ZNCC the input signals are centered around their respective means before computing cross-correlation. While this feature can account for changes in brightness in the input signals, maximization of the normalized dot product is still biased by high standard deviations. This behaviour is not displayed by NSQECC, as the criteria of minimizing squared errors produces the highest peak in the cross-correlation matrix when an exact match if found.

We further visualized the distribution of the biased vectors arising in ZNCC PIV. The map of Euclidean errors between the PIV predictions and the true translation reveals that biased interrogation volumes lay mostly (1) around the extraembryonic layers or (2) inside the gastrulating embryo, Figure S1b. Interrogation volumes in (1) mostly contain small patches of signal, e.g. a piece of nucleus, in close proximity to the borders of the interrogation volume. This is exemplified by the interrogation (red intesities) and search volume (blue intensities) shown in the top panel in Figure S1d. The small nucleus will be matched ambigously to all nuclei in the search volume, and the maximum peak will result when the small interrogation nucleus is aligned with the brightest nucleus in the search volume. We also observed that highly regular spatial patterns of the extraembryonic nuclei can lead to errors when using ZNCC, see the middle panel in Figure S1d. Likewise, these patterns lead to ambiguous cross-correlation peaks that are resolved to the brightest intensities. The biased vectors inside the gastrulating embryo, (2) are linked to diffuse patterns in the interrogation and search volumes. These patterns can be visualized in Figure S1d, bottom.

Overall, we conclude that NSQECC is more robust than ZNCC for analyzing biological data. We have also visualized and analyzed the intensity patterns that induce errors in ZNCC. The conflicting cases shown in the top and bottom panels in Figure S1d are consistent with the synthetic evaluation presented in Figure 2. Namely, these are analogous to the cases where synthetic volumes display low particle particle densities (top) and when the volumes contain diffuse intensity patterns due to high particle densities or large particles (bottom). We have found in our analysis that symmetry in biological samples can also induce biases in ZNCC.

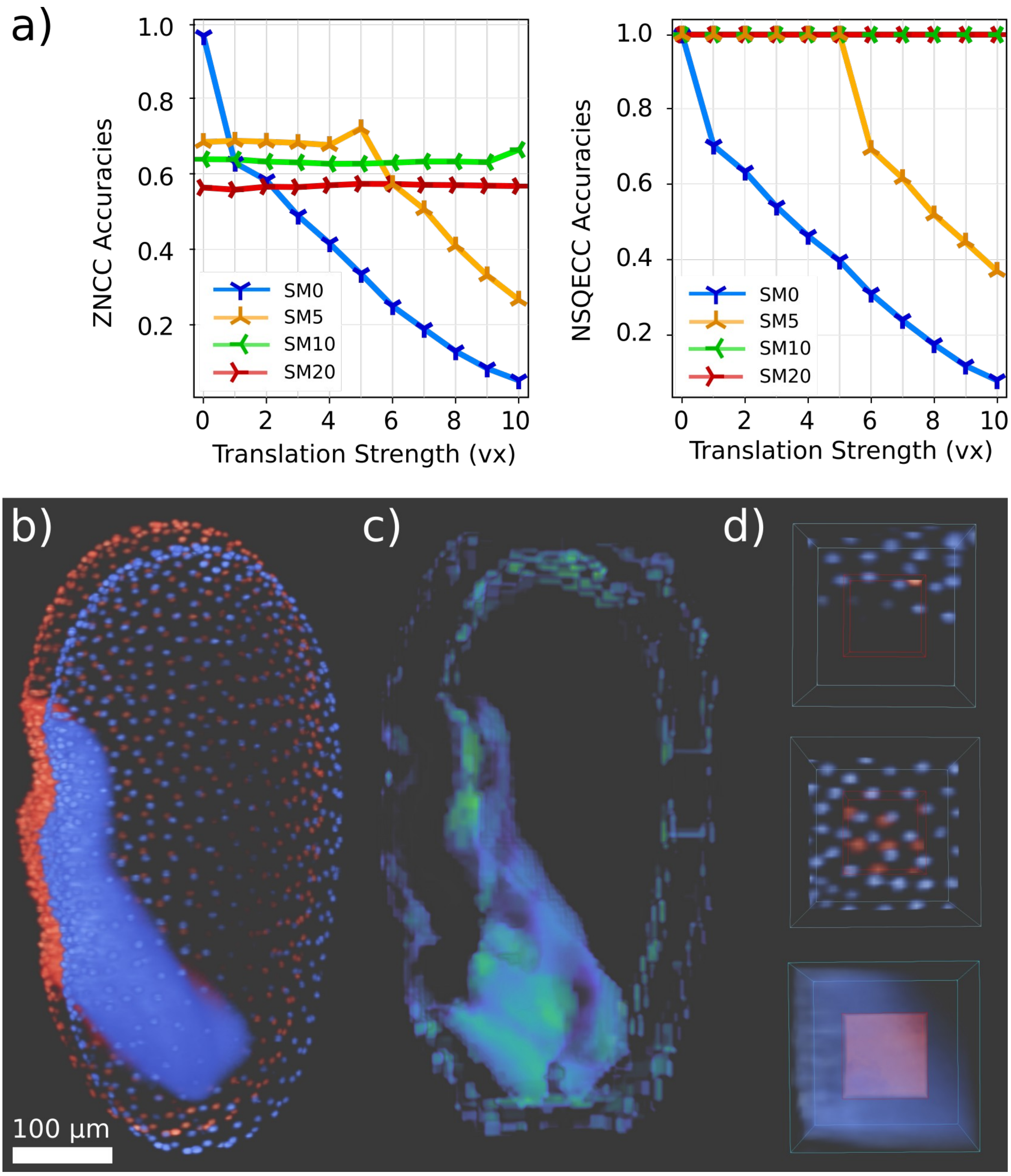

Supplementary Figure 1 Synthetic translations on biological data. a) Ratio of correctly computed PIV vectors with respect to the known underlying translation. A reduction of the maximum accuracy of ZNCC PIV analyses is observed as increasingly large search margins are introduced. NSQECC PIV converges to 100% accuracy as the search margin matches the underlying translation. b) Test volume (red) and a copy (blue) shifted by a translation of 10 voxels in each direction. c) Map of Euclidean errors obtained from the ZNCC PIV analysis between the test volume and a copy shifted by a translation of 3 voxels in each direction. d) Representative interrogation (red) and search (blue) volumes that lead to biases when using ZNCC on the *Tribolium castaneum* data set.

2-. Robustness of quickPIV to sub-optimal spatial resolutions

In order to speed up PIV analyses, we experimented with subsampling of the input volumes. Without subsampling, each 3D volume measures 1000x600x600 voxels. Furthermore, the data needs to be converted from 16-bit unsigned integer to float (32-bits) or double (64-bits), to avoid integer overflow during the computation of cross-correlation. This requires 2.8 (float) or 5.8 (double) Gibabytes of RAM to store the two input volumes to the PIV analyses. By subsampling the Tribolium castaneum volumes by a factor of 3, the size of the data is reduced to 334x200x200 voxels, which only requires 107 (float) and 214 (double) Megabytes of memory. In addition, the PIV parameters (interrogation size, overlap and search margin) are also down-scaled by the subsampling amount, leading to smaller interrogation and search volumes and faster cross-correlations.

Figure S2 shows the results from analyzing the same time-points of data set (i) analyzed in Figure 3, without previous subsampling of the input volumes. The vector fields shown in Figure 3 were obtained with a subsampling factor of 3. The high similarity between the results in Figure S2 and Figure 3 indicate that PIV analyses are robust to low spatial resolutions. This allowed us to resort to subsampling to dramatically speed up the execution times of the PIV analyses without significantly affecting the resulting vector fields. In particular, subsampling by a factor of 3 reduced the execution time by a factor of approximately 27, or 3^3 , from 29 minutes without subsampling to 55 seconds with a subsampling.

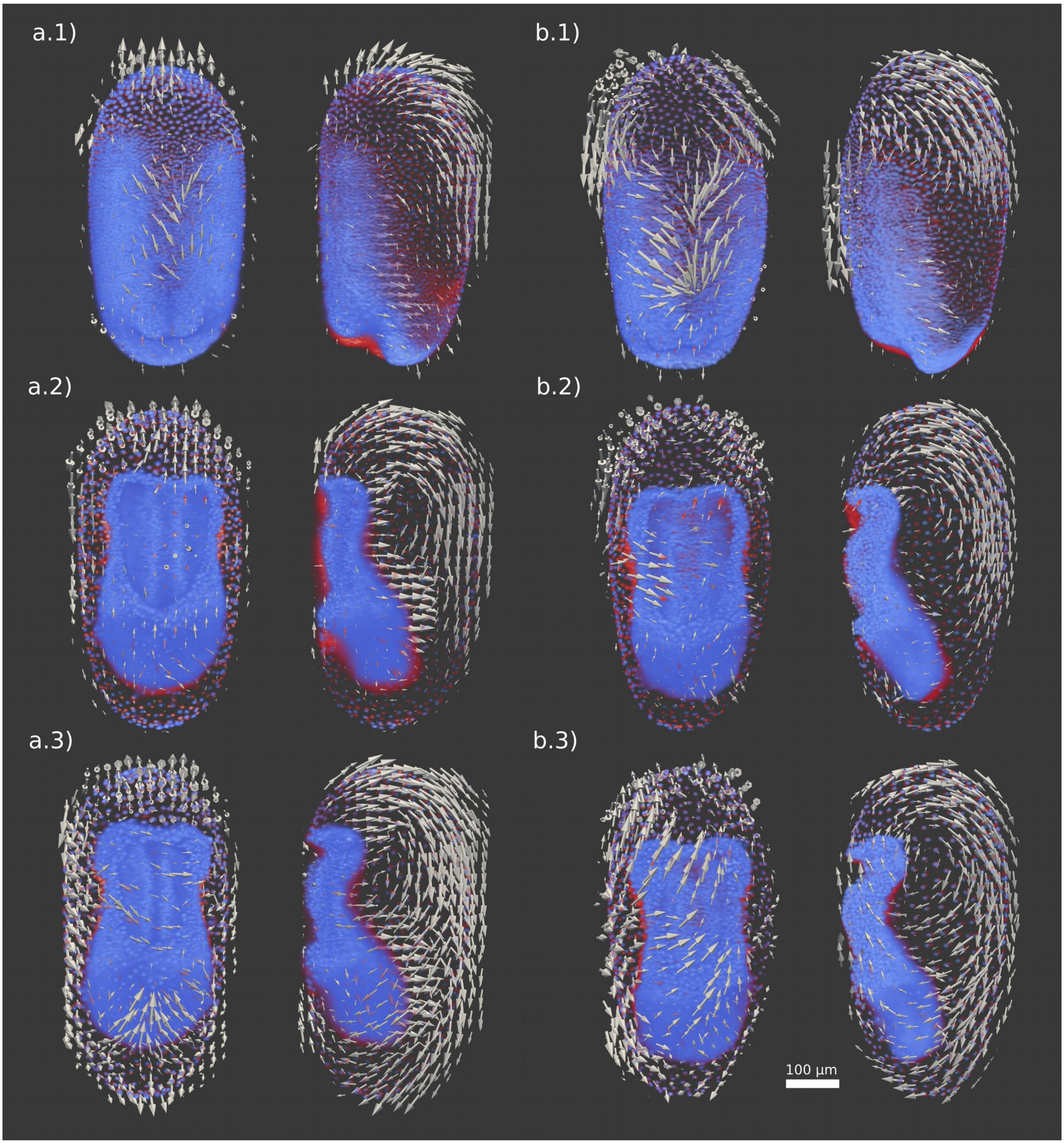

Supplementary Figure 2 PIV vector fields without subsampling. Three-time points of the two embryos in data set (i) are analyzed with quickPIV, and the resulting vector fields are plotted on top of the respective input volumes. The red intensities in each time-point indicate the fluorescence signal of the nuclear marker at the first time-point, while the blue colors correspond to the fluorescene signal at the second time-point. At each time-point, the embryos and the vector fields are shown from the ventral and lateral sides. The PIV parameters used for the PIV analyses of both embryos, a) and b), were: interrogation size of 60 voxels, search margin of 0 voxels, overlap of 30 voxels and multi-pass depth of 2 rounds. No subsampling of the input volumes was used. These analyses are analogous to the ones shown in Figure 3, where the input volumes were subsampled by a factor of 3 to reduce the time required for each analysis, and the PIV parameters were consequently reduced to interrogation size of 20 voxels, search margin of 0 voxels and overlap of 10 voxels.
